# The Role of Respiratory Microbiota in Lung Cancer

**DOI:** 10.7150/ijbs.51376

**Published:** 2021-08-25

**Authors:** Dan Wang, Jingyi Cheng, Jia Zhang, Fangyu Zhou, Xiao He, Ying Shi, Yongguang Tao

**Affiliations:** 1 Key Laboratory of Carcinogenesis and Cancer Invasion, Ministry of Education, Department of Pathology, Xiangya Hospital, Central South University, Hunan, 410078 China.; 2 NHC Key Laboratory of Carcinogenesis (Central South University), Cancer Research Institute and School of Basic Medicine, Central South University, Changsha, Hunan, 410078 China.; 3 Hunan Key Laboratory of Tumor Models and Individualized Medicine, Department of Thoracic Surgery, Second Xiangya Hospital, Central South University, Changsha, 410011 China.

**Keywords:** Respiratory System, Microbiota, Angiogenesis, Tumor

## Abstract

Recently, the impact of microorganisms on tumor growth and metastasis has attracted great attention. The pathogenesis and progression of lung cancer are related to an increase in respiratory bacterial load as well as changes in the bacterial community because the microbiota affects tumors in many ways, including canceration, metastasis, angiogenesis, and treatment. The microbiota may increase tumor susceptibility by altering metabolism and immune responses, promoting inflammation, and increasing toxic effects. The microbiota can regulate tumor metastasis by altering multiple cell signaling pathways and participate in tumor angiogenesis through vascular endothelial growth factors (VEGF), endothelial cells (ECs), inflammatory factors and inflammatory cells. Tumor angiogenesis not only maintains tumor growth at the primary site but also promotes tumor metastasis and invasion. Therefore, angiogenesis is an important mediator of the interaction between microorganisms and tumors. The microbiota also plays a part in antitumor therapy. Alteration of the microbiota caused by antibiotics can regulate tumor growth and metastasis. Moreover, the microbiota also influences the efficacy and toxicity of tumor immunotherapy and chemotherapy. Finally, the effects of air pollution, a risk factor for lung cancer, on microorganisms and the possible role of respiratory microorganisms in the effects of air pollution on lung cancer are discussed.

## Introduction

The human microbiome is a complex ecosystem primarily composed of a large number of various bacteria, viruses and fungi. The metagenome is even greater than the human genome by 150 times. These microorganisms mainly settle in human skin and mucous membranes, among other sites, and coevolved with humans 1.

In addition to inflammatory diseases, microorganisms have also been confirmed by many epidemiological studies to be related to many noninflammatory diseases, such as obesity, diabetes, and tumors. The types of microorganisms carried by different people are very diverse, and they are dynamically affected by external factors such as body part, diet, antibiotics, and lifestyle 2. Meanwhile, the microbiome also affects the host's nutrition, immunity, metabolism and the occurrence or development of diseases including cancer.

At present, it is a good time and a key turning point for human microbiome research. The development of DNA sequencing technology, especially 16S rRNA gene sequencing and the related transcriptome, proteomics, metabolism, and immunological analysis technologies, helps us to determine the changes in the microbiome in health or various diseases more accurately. Based on many studies of different microbiomes in healthy populations or disease states, investigations have gradually transitioned to explore the mechanism.

The carcinogenesis of *Helicobacter pylori (H. pylori)*, which promotes chronic inflammation or host DNA damage through molecules such as CageA, VacA and NapA2, is assured. However, interestingly, *H. pylori* infection lowers the risk of esophageal adenocarcinoma and is a strong protective factor 3. However, in addition to the carcinogenesis of *H. pylori* and chronic inflammation, few studies have convincing evidence of other gastric microbiomes in the pathogenesis of cancer. For colorectal cancer, *Fusobacterium nucleatum* (*F. nucleatum*) increased the risk, and in subsequent animal and laboratory experiments, *F. nucleatum* was verified to increase cancer risk through attachment and invasion mechanisms and tumor immune escape 4, 5. Tumors are not only related to microbial imbalances in the microenvironment next to the tumor, whose classic example is liver cancer induced by hepatitis B virus and cervical cancer induced by human papilloma virus (HPV), but the microbiota in different parts may also affect each other. The intestinal microbiota and metabolites have been confirmed to promote the development of tumors in regions that are far away from the intestine, such as the liver and prostate 6, 7.

In the lung, which was once considered sterile, the existence of microbiota different from the upper respiratory tract has also been demonstrated 8. The lung microbiome may partially explain why the lung is an important organ for tumor formation and/or metastasis. The inflammatory and immune responses caused by the lung microbiota affect lung tumorigenesis. Lung bacteria stimulate tissue-resident γδ T cells, promoting the infiltration of neutrophils and the proliferation of tumor cells 9. Different changes in the immune system caused by the microbiome may lead to different changes in tumor progression. Tumor-associated macrophages (TAMs) are alveolar macrophages (AMs) infiltrated into tumor tissue. The two phenotypes M1 and M2 of TAMs have different effects on lung cancer. The M1 phenotype plays a role in killing tumor cells, while M2 macrophages make the tumor microenvironment conducive to tumor progression 10. The lung microbiome has become an important object for the study of tumorigenesis and development.

Similar to other solid tumors, the growth and metastasis of lung cancer requires oxygen, nutrient replenishment, and transportation of metabolic wastes, which require angiogenesis. An increase in microvessel density can be observed in early lung cancer, which may confirm this point. Studies in recent years have found that the lung is also a hematopoietic organ, and a large number of megakaryocytes capable of producing platelets can be found in the lungs of mice 11. At the same time, the role of platelets in promoting angiogenesis has also been demonstrated 12.

## The relation between respiratory microorganisms and lung cancer

The respiratory system is an important part of the human body connecting the outside world with the body. Complex and diverse microbial communities in the respiratory tract 13 play unique roles in biological processes. Respiratory microorganisms are of great importance in a variety of diseases. Studying the structure of human respiratory microorganisms and their correlation with disease progression is conducive to clarifying the mechanism of respiratory and other systemic diseases, which is of great importance in prevention and treatment. The pathogenesis of lung cancer is related to changes in bacterial burden and composition in the respiratory system 9. At present, research on the correlation between respiratory microorganisms and lung cancer is still in its infancy, but it has already been able to explain some aspects of the disease.

### Respiratory bacteria and lung cancer

The respiratory system consists of the respiratory tract and lung. There are fixed bacteria in the respiratory system of healthy people 14. Respiratory microecology will change dramatically under the condition of disease, which is responsible for the occurrence and development of disease 15. The pathogenesis of lung cancer is a process involving the interaction of many factors, including dynamic changes in respiratory microbial structure. Therefore, the improvement of bacterial structure in the respiratory tract has the potential to regulate the development of cancer, is important for research and thus is valuable to apply.

### Bacteria in respiratory tract and lung cancer

In the healthy respiratory tract, *Proteobacteria*, *Firmicutes*, *Fusobacteria* and *Bacteroidetes* are the main phyla and classes, and *Pseudomonas*, *Streptococcus*, *Prevotella*, *Veillonella*, *Haemophilus* and *Neisseria* are the main genera of flora 14, 16.

There are structural changes in the respiratory tract flora in the case of lung cancer. The correlation between respiratory tract flora and the pathogenesis of lung cancer has previously been revealed. In patients with lung cancer, multiple anaerobic bacteria exist in the lower respiratory tract 17, represented by *Actinomyces* and *Peptostreptococcus*. There is also a large quantity of oral bacteria, such as *Streptococcus* and *Wechsler*, which are related to the upregulation of the ERK and PI3K signaling pathways 18. Moreover, both *Mycobacterium tuberculosis (M. tuberculosis)* 19 and *H. pylori* 20 infection take part in promoting the development of lung cancer. Compared with adjacent normal tissues, the contents of *Modestobacter* in lung cancer tissues are higher, but *Propionibacterium* and *Enterobacteriaceae* are lower 21. A lower airway microbiome analysis showed that *Bradyrhizobium japonicum* could only be detected in lung cancer patients, but the genus *Acidovorax* could be detected in not only patients with lung cancer but also patients with benign lung disease 22. Moreover, the numbers of *Eubacterium xylanophilum*, *Eubacterium eligens* and *Clostridium* in the human respiratory tract tend to have a positive correlation with the occurrence of small cell lung cancer (SCLC), and the number of *Prevotella* and *Pseudobutyrivibrio ruminis* may have a negative correlation with the occurrence of SCLC 23.

Various microorganisms form a complex regulatory network linked with lung cancer. Different microorganisms in the same type of lung cancer or the same microorganisms in different types of lung cancer may play different roles. Additional elaboration of the detailed regulatory network of microorganisms in lung cancer is expected.

### Pulmonary flora and lung cancer

The opinion of the aseptic lung has been repudiated. Many studies have found that the dominant populations in healthy lungs of adults are *Firmicutes*, *Bacteroidetes*, *Proteobacteria*, *Actinobacteria*, and *Fusobacterium* 24, 25. Furthermore, there are also related findings that for healthy adults, the lung microbiome has no significant difference in spatial space 25. In terms of diversity analysis, there was no significant difference in beta diversity between nonmalignant lung tissue and tumor lung tissues, but the alpha diversity of the microbial group was significantly reduced in tumor lung tissues 26.

Lung microorganisms are closely related to certain lung diseases and health because microbiome dysbiosis may increase tumor susceptibility by changing metabolism, altering the immune response, promoting inflammation, and increasing toxic effects 27. Therefore, defining the composition and changes of the microbial group in lung cancer patients is conducive to deepening the understanding of the mechanism of lung cancer. At the same time, this knowledge may provide new ideas for preventing lung cancer or treatment intervention. For example, the epidemiological relationship between *M. tuberculosis* and lung cancer can be explained by the mechanism of chronic inflammation-associated carcinogenesis 28.

The results of an experimental analysis of contralateral bronchoalveolar lavage fluid (BALF) samples via 16S rRNA sequencing showed that the number of operational taxonomic units (OTUs) was significantly different between the lung cancer group and the benign mass-like lesion group, while β-diversity was still no different. The Chao 1 and Shannon indices in the lung cancer group were higher than those in the normal group. Among the five detected phyla and seven detected genera, the phyla with increased relative abundance were *Firmicutes* and *TM7* in the lung cancer group while the most abundant genera were *Eillonella*, *Megasphaera*, *Atopobium* and *Selenomonas*. The abundance of *Atopobium* and *Selenomonas* decreased in both groups 29. These changes may suggest a way to affect lung cancer by altering the pericancerous microenvironment.

In addition, the proportion of *Firmicutes*/*Bacteroidetes* in the lung cancer group with smoking was significantly higher than that in the lung cancer group with nonsmoking, and the relative abundance of some bacteria was different from that in the nonsmoker group. Meanwhile, increased *TM7* in both chronic obstructive pulmonary disease (COPD) and lung cancer suggests that *TM7* is likely to promote the progression of COPD to lung cancer. Finally, *Megasphaera* and *Veillonella* were identified as biomarkers for lung cancer, meaning they could be used to predict lung cancer or as microbial therapeutic targets 29.

Another study collecting saliva and BALF found that the microbial alpha diversity in lung cancer patients was low, and this difference from the control group was not related to the sampling site. However, certain bacterial groups associated with lung cancer are affected by the sampling site and pathological type, and the same is true for the metabolic differences of the microbial population. Via KEGG metabolic pathway enrichment analysis and functional analysis, activated and enriched behaviors usually include antibiotic resistance and sorting, folding and degradation as well as metabolism of amino acids and glycans. However, regardless of the sampling site, the microbiota composition differed between the two groups. Finally, the study also suggests that the good bacterial biomarkers in BALF are *Treponema* and *Filifactor* and that *Filifactor* has good resolution in distinguishing between healthy controls and lung cancer patients 30.

However, symbiotic microbial biomarkers of lung cancer or microbial therapies still require further large-scale studies.

### Respiratory system fungi, viruses and lung cancer

A retrospective study suggested that pulmonary lymphoepithelioma-like carcinoma is closely related to Epstein-Barr virus (EBV) infection, and EBV latent infection is the main state of pulmonary lymphoepithelioma-like carcinoma 31. Among EBV-encoded small nonpolyadenylated RNA (EBER)-positive pulmonary lymphoepithelioma-like carcinoma (LELC) cases, latent membrane protein 1(LMP1) and viral capsid antigen (VCA) are expressed in approximately half and a quarter of these cases, respectively. Many recent related studies confirm that lymphoid-specific helicase (LSH) can affect the occurrence and development of non-small cell lung cancer (NSCLC) in cancer metabolism and epigenetics through different mechanisms or as intermediate products. For example, Go, Ichi, Nii, and San complex subunit 4 (GINS4) is highly expressed in NSCLC and contributes to tumorigenesis. LSH increases GINS4 expression by binding to the 3'-untranslated region 32. Moreover, LSH promotes lipid metabolism-related genes and becomes a vital inhibitor of ferroptosis in carcinogenesis *in vitro* or *in vivo*. The increased expression of LSH is regulated by EGLN1 and c-Myc 33. The overexpression of LSH in nasopharyngeal carcinoma is linked to EBV infection, and LSH becomes a cancer driver by suppressing the activity of fumarate hydratase 34, 35.

A clinical study across Taiwan showed that a high risk of lung cancer involved being exposed to influenza and that there was more risk after cumulative exposure to flu. However, the mechanism is currently unknown 36. In samples from patients with lung adenocarcinoma, the prevalence of John Cunningham virus (JC virus) and whether virus was present in metastatic lymph nodes were evaluated, suggesting that JC virus may be involved in lung cancer 37.

The data show that the incidence of lung cancer in human immunodeficiency virus (HIV)-infected patients is higher than that in uninfected patients. The cumulative incidence of lung cancer among HIV-infected groups continues to rise. In addition to a reduction in all-cause mortality, immunosuppression, pulmonary inflammation, and systemic inflammation are all specific risk factors for increased risk 38. There are studies that suggest that direct viral oncogenesis is a mechanism of lung cancer in HIV infection, but there is no exact support for direct HIV oncogenesis in the lungs. Only some early studies have shown some direct oncogenic activity among HIV virus proteins. Studies evaluating antiretroviral treatment did not lead to a higher risk of cancer. In addition, the hypothesis that P450 cytochrome inhibitors may affect the metabolism of lung cancer carcinogens has not been found in The French Hospital Database 39.

In short, the types and quantities of human microorganisms are very large. Although the Human Microbiome Project (HMP) does not use the lung as an organ of research, the traditional view that the lung is sterile has been overthrown, and the study of the microbiome in the lung has developed rapidly as a new field. There are still many areas worth exploring.

### Lung cancer-related microorganisms in other tumors

The carcinogenesis of *H. pylori*, through Helicobacter pylori toxin such as CageA, may also be a way for *H. pylori* to induce lung cancer 40, 41. HPV is not only related to cervical cancer and breast cancer but is also a risk factor for lung cancer 41, 42.

Clearly, a healthy commensal microbiome is tightly related to immune cell functioning. The study of Rea Bingula et al. on the gut-lung axis mainly used the gut as an example and tracked the effects of intestinal immune stimulation on the lung to speculate that the situation originating from the lung mucosa and lymph nodes is also similar 43. The two “cooperate” and influence each other.

The lung cancer-related microorganisms summarized above have also been found in other tumors.

Alterations in these changing microorganisms in the microenvironment of other tumors have also been reported by other papers. For example, *Atopobium* may not only lead to a high risk of esophageal squamous cell carcinoma 44 but also increase the risk of endometrial cancer in the uterus 45. *Firmicutes* was found to be more abundant in the urine of bladder cancer patients, and *Veillonella* was more abundant in the healthy control group than in the bladder cancer group 48. *Filifactor* in the oral cavity is positively related to oral squamous cell carcinoma 126, 127 and is overrepresented in the tongue coating of pancreatic head carcinoma patients 128. *Selenomonas* is overrepresented in the stool of colorectal cancer patients 129 and is more abundant in left colorectal cancer patients compared to right colorectal cancer patients 130.

The high risk of esophageal squamous cell carcinoma may be related to *Atopobium*, a kind of microorganism that is reduced in lung cancer 44. An *in vitro* experiment found that *Atopobium vaginae* (which becomes less abundant in lung cancer) induces proinflammatory cytokine expression in endometrial cells and may lead to significant endometrial cancer if *Porphyromonas somerae* also exists 45.

In addition,* Propionibacterium* in the lung is negatively related to lung cancer, while *Propionibacterium* in the prostate is positively correlated with prostate cancer 46, 47. For bladder cancer, the most abundant phylum in both groups was *Firmicutes,* which became more abundant in lungs with lung cancer than in healthy lungs. However, *Veillonella*, suggested as a lung cancer marker, is more common in healthy urine 48.

The progress and relationship of lung cancer-related microorganisms and other tumors needs further research and discovery.

## The influence of pulmonary microorganism on lung cancer

At present, the understanding of the influence of the microbiota on tumors is still insufficient. The influence of microorganisms on tumors involves many aspects, such as canceration, angiogenesis, and therapy.

### The possible mechanism of the influence of pulmonary microorganisms on lung cancer canceration

In the process of cancer occurrence, microecology deviates from the normal steady-state structure and gradually forms the microenvironment of promoting cancer to induce a series of chain reactions, finally causing canceration. An imbalance in the lung microecology may affect the canceration of lung cancer through inflammation, immunity, metabolism and genotoxicity.

### Inflammation

Chronic lung inflammation is a critical risk factor for lung cancer 49, and proinflammatory cytokines, adhesion molecules, and growth factors can provide a favorable microenvironment to promote the survival and proliferation of tumor cells 50. Microbial organisms are confirmed to be important causative agents of cancer-inducing inflammation 51.

Membrane receptors play a vital role in cancer caused by microorganisms through the process of inflammation. Membrane receptors such as pattern-recognition receptor (PRR), cluster of designation (CD), and TLR proteins can recognize microorganisms and their production, proinflammatory cytokines and some other signaling molecules, leading to effects on apoptosis and cellular proliferation 52. In COPD patients, infection with nontypeable *Haemophilus influenzae* (NTHi) can induce epithelial cells to express IL-17C and neutrophils to promote tumors 53. Interleukin-6 (IL-6) also exerts a significant function in lung cancer by inducing a COPD-like inflammatory response 54.

### Immunity

A small number of cancer cells in the body can be eliminated by the antitumor immune mechanism, which may malfunction with the growth of cancer cells 55. The regulation of the cancer immune response by microbiota has also received more attention.

Commensal bacteria are involved in maintaining immune homeostasis against cancer through γδT17 responses 56. Lung immunity after antibiotic treatment tends to be defective, thus creating a more favorable environment for the occurrence of cancer. However, another study suggested that the local microbiota could activate lung-resident γδ T cells to promote inflammation associated with lung adenocarcinoma and tumor cell proliferation 9. Commensal bacteria also regulate the antitumor response in the lung through alveolar macrophages, maintaining their low level of CCL24 secretion 57. Although it is unknown and difficult to define whether the immune environment is regulated by specific bacteria with dedicated functions in the lung, the role of some bacteria has been proposed. *Morganella morganii* and *Escherichia fergusonii*, two ubiquitous opportunistic pathogens from the *Proteobacteria* phylum in the lung, can produce different virulence factors to exert an immunostimulatory effect 58. NSCLC patients react to *Streptococcus salivarius* and *Streptococcus agalactiae* with significantly higher frequencies of T helper type 17 (Th17) and Th1 cells than healthy controls 59. Recently, a study revealed that *Pasteurella* has a positive correlation with cytotoxic CD8+ TILs and a negative correlation with M2 macrophages. There is a significant positive correlation between *Coriobacteriaceae* and M2 macrophages and a negative correlation with CD8+ cells 60. Together, these immune reactions affect the occurrence and development of lung tumors.

These results suggest that the microbiota in the respiratory system has an important influence on the occurrence of lung cancer, which may establish a permissive environment for lung cancer.

### Metabolism

Microorganisms have been proven to participate in regulating host metabolism, detoxification, vitamin levels and nutritional status 61.

Changes in microorganisms are related to the production of carcinogens such as acetaldehyde 62 and deoxycholic acid 63. This metabolic imbalance may result in the formation of carcinogens in the lung or lead to the secondary processing of foreign carcinogens. The lung microbiota is considered to be connected with specific insults related to lung cancer via CD36, which regulates the treatment of cyanobacteria-derived microcystin residues in the lung alveoli, upregulating the expression of poly (ADP-ribose) polymerase 1 (PARP1) 64.

Microorganisms may also play an anticancer role by influencing metabolism. Short-chain fatty acids, as products of intestinal bacteria, exert anti-inflammatory and antiproliferation functions 65. Moreover, butyrate treatment could lead to an upregulation of miR-3935 and an inhibition of the proliferation and migration of A549 cells 66.

### Genotoxicity

Host DNA damage can cause cell death and affect the expression of tumor suppressor genes or oncogenes. Some bacterial molecules have been revealed to be related to genotoxicity. For instance, *Bacteroides fragilis* toxin and *Escherichia coli* toxin have been found to be related to inducing double-stranded DNA damage 67, 68. Chemicals produced by bacteria such as reactive oxygen species (ROS) produced by *Porphyromonas*, hydrogen sulfide produced by *Clostridium cholephilum* and superoxide dismutase produced by some bacteria are also responsible for genomic instability 69-71, increasing the probability of cancer. In addition, microorganisms can also play a carcinogenic role through the integration of their DNA and host genome.

### The possible mechanism of the influence of pulmonary microorganisms on the metastasis of lung cancer

Microorganisms also play an irreplaceable role in the metastasis of lung cancer. Oral taxa that are rich in the lower airway of lung cancer patients, such as *Streptococcus* and *Veillonella*, promote the proliferation, invasion and metastasis of lung cancer cells by activating the ERK and PI3K pathways in airway epithelial cells 18. Ubiquitin molecules in host cells can activate protein tyrosine phosphatase A (PTPA) secreted by *M. tuberculosis*, which promotes the proliferation and migration of the human lung adenocarcinoma cell line A549 72. Additionally, the metastasis of lung cancer can be affected by microorganisms through inflammatory mechanisms. The stimulation of gram-negative bacteria with TLR4, a receptor that mediates inflammation, increases adhesion, migration, and metastatic spreading of NSCLC cells 73. Similarly, gram-positive pneumonia augments NSCLC metastasis via TLR2 activation 74. Inflammation also causes crosstalk between bacteria and tumor cells through quorum sensing peptides to promote tumor metastasis 75. In terms of immunity, a decrease in bacterial load is linked with reduced regulatory T cells and enhanced T cell and natural killer (NK) cell activation, which contributes to a resultful reduction in lung metastases 58.

### Angiogenesis is an intermediary between microorganisms and tumors

Human microbiota can affect angiogenesis 75, 76. An important possible reason is that inflammatory factors related to microbiota-induced inflammation affect angiogenesis. Angiogenesis has an effect on infection, tumors and other pathological processes. In recent decades, many scholars have studied how angiogenesis affects tumor growth and metastasis. There are many clinical data to prove that antiangiogenic drugs are effective in cancer patients. Therefore, the effect of the microbiome on angiogenesis makes it possible to manipulate the human microbiome to change angiogenesis in the disease process.

**Figure 2:**
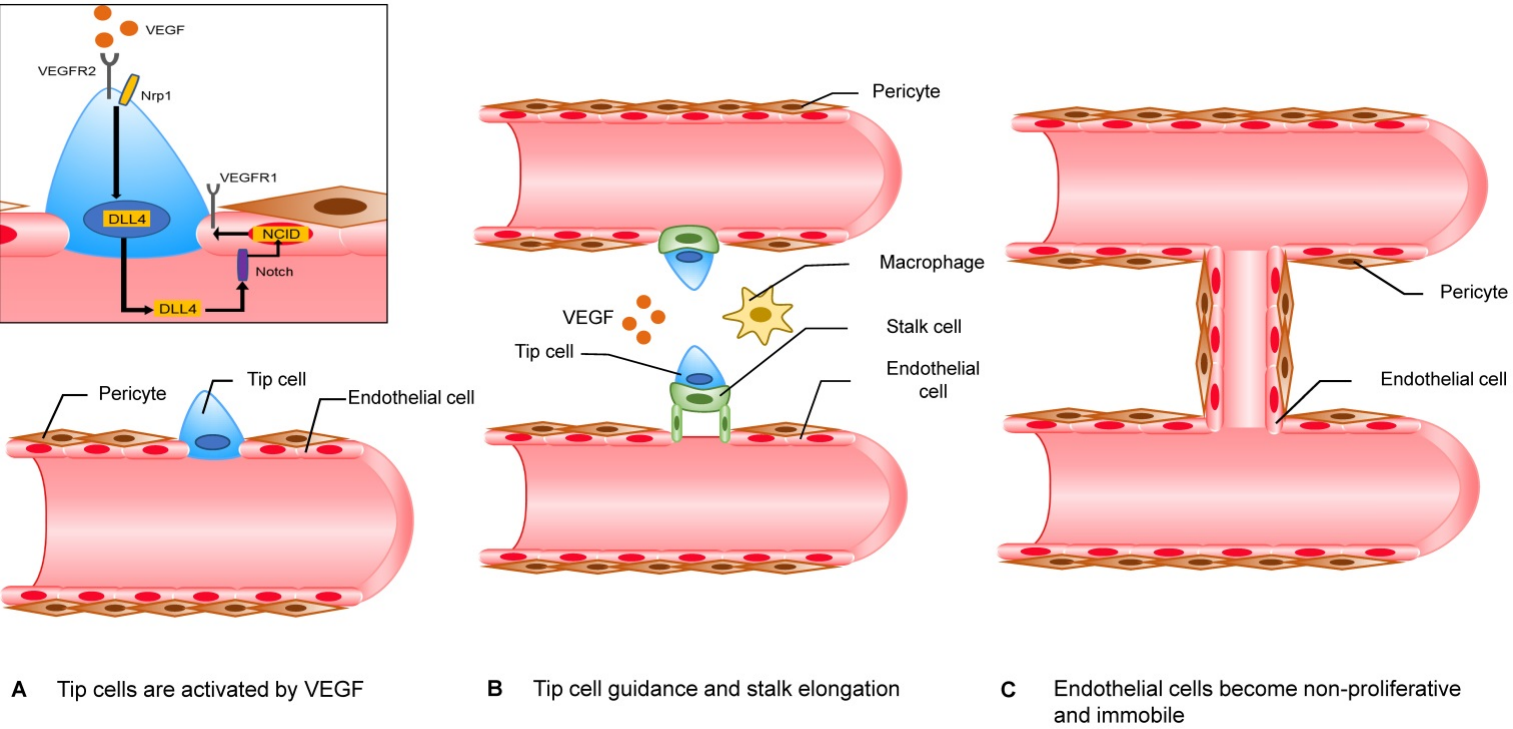
** The mechanism of sprouting angiogenesis.** The “tip cells” at the forefront of the angiogenic sprout migrate along the angiogenic signal. “Stalk cells” continue to proliferate behind “tip cells”. However, the classification of “tip cells” and “stalk cells” is not fixed and depends on the ratio of VEGFR1/VEGFR2 on the cell membrane. ECs that express more VEGFR1 have lower reactivity to VEGF, while ECs that express more VEGFR2 have higher reactivity to VEGF. The specific mechanism is as follows. EC with a higher VEGFR2 ratio binds to the receptor Notch of the adjacent EC through its expressed DLL4, which can promote the expression of NICD by the adjacent EC. NCID can increase the expression of VEGFR1 and decrease the expression of VEGFR2. When adjacent "tip cells" meet, the two angiogenic sprouts fuse into a branch with the help of macrophages. Once the branch is perfused, ECs lose their ability to proliferate and to migrate. After recruiting enough pericytes, a mature blood vessel is formed.

Accepted mechanisms of neovascularization include sprouting angiogenesis, vasculogenesis, vasculogenic mimicry and vascular intussusception, and the above four mechanisms are thought to act on pathological neovascularization at the same time. Among them, sprouting angiogenesis models have been extensively studied (Figure 2). To avoid necrosis that is due to hypoxia, cells in hypoxic regions secrete vascular endothelial growth factors (VEGF). VEGF promotes endothelial cells (ECs) to secrete matrix metalloproteases (MMPs), which can degrade their basement membrane (BM) and extracellular matrix (ECM) 77. BM is located on the basal surface of ECs and has a supporting, connecting and fixing effect on endothelial cells. When the stability of BM is disrupted, endothelial cells gain the ability to proliferate and to migrate. In this model, there are two important types of cells, namely, “tip cells” and “stalk cells.” “Tip cells” migrate under the stimulation of angiogenic signals. They are the foremost cells. The cells after “tip cells” are called “stalk cells” and have a strong ability to proliferate. Similar to their name, these cells are comparable to the stalk of an angiogenic sprout. When adjacent “tip cells” meet, two angiogenic sprouts fuse into a single branch. Once the branch is perfused, ECs lose their ability to proliferate and to migrate. After recruiting enough pericytes, a mature blood vessel forms 78.

Microorganisms participate in the abovementioned angiogenesis mechanism through VEGF, ECs, inflammatory factors and inflammatory cells. Irradiated mice treated by fecal microbiota transplantation show increased expression of VEGF, promoting angiogenesis 79. The immune response to bacteria *in vitro* and in a sepsis mouse model alters angiogenesis 80. Alteration of intestinal microbiota was observed in mice fed stool from colorectal cancer patients, with enhanced expression of genes associated with angiogenesis and accelerated tumorigenesis 81. Positive effects of specific microorganisms on tumor angiogenesis have been previously identified. In the CCSPcre/K-rasG12D mouse lung cancer model exposed to NTHi lysate weekly, the infiltration of Th17 cells in the lung tissue increased, with tumor growth accelerating 82. The Th17 cell population is capable of producing IL-17. IL-17 can increase angiogenesis by inducing the generation of multiple angiogenic mediators, angiogenic chemokines, and cytokines that promote angiogenesis 83-85, which suggests that NTHi-induced Th17 cells may promote lung cancer development by promoting angiogenesis. Another link to tumor vascularization of carcinoma of the lungs is *H. pylori* through the assumption of direct damage and chronic inflammation, and *H. pylori* multiplication induces a systemic immune response coupled with the host's susceptibility and may induce lung cancer 86. Helicobacter pylori VacA (exotoxin) exists in the lung and can induce lung cells to generate IL-8 and IL-6. Vac has cytotoxic effects and can specifically act on airway epithelial cells to induce interleukins 87. IL-8 is a promoting factor for tumor angiogenesis; therefore, *H. pylori* in the lung is closely connected with tumor vascularization of lung carcinoma. HPV can also significantly enhance the angiogenesis of lung cancer cells *in vitro* 88, which may correlate with the PI3K/Akt signaling pathway and c-Jun 89.

Microorganisms that inhibit tumor vascularization have also been confirmed. The fungi zj-14, zj-17 and zj-36 can significantly inhibit HPV-16E7-stimulated microtubule formation 90. Therefore, they may ultimately suppress the angiogenesis of lung carcinoma by suppressing the angiogenic effect of HPV on lung cancer. Moreover, *A. muciniphila* and *E. hirae* induce dendritic cells to secrete interleukin-12 (IL-12) 91, which can inhibit tumor angiogenesis 92. This action may be one of the mechanisms by which the microbiota exerts tumor suppressive effects by affecting angiogenesis. Therefore, microorganisms are indeed closely related to tumor angiogenesis, which will also be an important direction for future research. It is worth noting that different types of tumors in various sites have different microenvironments. The mechanisms by which different microorganisms influence angiogenesis in cancer are also worth comparing.

### The effect of vascularization on tumors

Tumor angiogenesis plays a significant role in tumor initiation, dormancy, patterned advancement and diversion.

In addition to maintaining tumor growth in the primary site, tumor angiogenesis can also promote tumor metastasis and invasion, thereby affecting the formation of secondary tumor foci. VEGF has a vital impact on neoplasm endotheliocytes including upregulating the production of proteases, promoting degradation of the basilar membrane, and prompting the expression of molecules that connect neoplasms with endotheliocytes 93.

Matrical cells, except endotheliocytes, are involved in angiogenesis-driven diversion processes, such as abnormal pericytes in structure or number. This process causes the cell gap to become larger, the tumor cells passively to leak out, and then to metastasize 94.

Therefore, an increased level of VEGF, a vital angiogenic factor produced in angiogenesis, is related to an unfavorable outcome in NSCLC patients 95, 96.

### The uniqueness of blood supply to lung cancer

There are four known models of blood supply to lung cancer: basal, nipple, diffuse, and alveolar blood vessel dependent. The fourth blood supply mode of lung cancer is exactly the difference between lung cancer and other tumors. There is no substantial damage in this model. The lack of tumor-related stroma and new blood vessels indicates that tumors do not undergo angiogenesis. We found that the tumor was full of alveoli, so it can be seen that lung cancer depends on the original alveolar blood vessels for blood supply 97.

### The role of the microbiota in tumor therapy

The microbiota has also played a part in antitumor therapy. Aerosolized antibiotics can reduce the lung microbiota, which is associated with a decrease in regulatory T cells and a reduction in the growth of melanoma B16 lung metastases 58. Possible therapy targeting the respiratory microbiota has also been found in the therapy of primary lung cancer. In a genetically engineered lung adenocarcinoma mouse model, mice treated with antibiotics showed decreased cancer progression. Reduced lung bacterial load is associated with diminished tumor burden 9. In NSCLC patients, clarithromycin treatment is related to prolonged survival and increased benefit, which is associated with decreased IL-6 levels. Its antitumor effect may be related to regulating the microbiome and reducing the release of cytokines that promote inflammation 98.

In recent years, immune checkpoint blockers (ICBs) have shown great development potential in tumor therapy. Two targets, cytotoxic lymphocyte antigen-4 (CTLA-4) and programmed cell death protein 1 ligand 1 (PD-L1), have drawn great attention. As a critical regulator of the immune system, the microbiota influences the effect of immunotherapy. *Bacteroidales* plays a role in the antitumor effects of CTLA-4 blockade 99. The relationship between *Bifidobacterium* and the efficacy of PD-L1 antibody therapy has also been reported in which enhanced function of dendritic cells is involved 100. However, an abnormal or unfavorable microbiome composition can lead to primary resistance and unsatisfying clinical outcomes 101, 102. Moreover, carcinoma-related microbiota has inspired specific targets of immunotherapy. The continual presence of EBV in nasopharyngeal carcinoma promotes vaccine trials of specific epitopes (such as LMP1 and LMP2) and the transfer of certain cytotoxic T lymphocytes 103.

The microbiota has also become nonnegligible to maximize the efficacy and to minimize the toxicity of tumor chemotherapy. The microbiota can cause an increase in the level of ROS produced by myeloid cells, which can promote cancer cell death induced by oxaliplatin 104. The anticancer efficacy of cyclophosphamide is also closely related to the alteration of the microbiota and the resultant immune response 105. Despite the capacity, the microbiota also affects drug resistance and chemotherapy toxicity. *Gammaproteobacteria* can mediate tumor tolerance to gemcitabine through the long isoform of cytidine deaminase 106, while the modulated autophagy pathway is related to chemoresistance mediated by *Fusobacterium (F.) nucleatum* 107. Diarrhea, a common side effect caused by irinotecan, is correlated with bacterial β-glucuronidase, which can regulate the bioactive form of irinotecan 108.

However, current studies mainly focus on the intestinal microbiota, but the intestinal and respiratory microbiota differs in many ways, such as composition and function. The difference between the role of the respiratory microbiota and intestinal microbiota in tumor therapy deserves more research, especially on the influence of local microenvironment changes in lung cancer during therapy.

## The microbiota in the effect of air pollution on lung cancer

The relationship between the microbiota and lung cancer may explain some lung cancer risk factors that affect the respiratory microbiota, such as air pollution.

### Air pollution, the microbiota and lung cancer

Air pollution induces alterations in the respiratory microbiota. Exposure to biomass smoke led to elevated bacterial abundance as well as diversity in rat lungs 109, but bacterial diversity was reduced after exposure to particulate matter 2.5 (PM2.5) in the mouse respiratory tract 110, which suggests that the specific components of the pollutants and the response of different species to pollutants need to be considered to draw a conclusion. There were differences in the oropharyngeal microbiota at different levels of air pollution among healthy young subjects from northeastern China 111. The relative abundance of *Actinobacteria* and* Firmicutes Proteonacteria* was higher in samples from polluted areas, with* Bacteroidetes* and *Fusobacteria* being significantly lower. Domestic biomass fuel combustion can cause household air pollution (HAP). The abundance of *Streptococcus* and* Neisseria* was higher in the high HAP-related particulate exposure group than in the low-exposure group 112. Regarding microbial activity, benzo[a]pyrene (B[a]P) altered the transcriptome of the human gut microbiome but had no significant impact on the microbiome composition 113.

Epidemiological studies clearly demonstrate that air pollution is related to the morbidity and mortality of lung cancer 114-117. Exposure to PM2.5 induced an increase in tumor nodules in tumor-bearing mice, with elevated levels of 12 angiogenesis factors and a perturbed microecosystem of the upper respiratory tract 118. However, there are difficulties in confirming the causal relationship between changes in the microbiome and changes in tumor development after air pollution exposure.

### The microbiota and the effects of air pollution on lung cancer

Air pollution has an impact on the occurrence of lung cancer from multiple aspects related to various biochemical pathways. Genotoxicity plays a vital role in the impacts of air pollution on lung cancer. DNA oxidative damage is related to the initial stage of carcinogenesis. The premutagenic lesion 8-hydroxy-2'-deoxyguanosine (8-OHdG) is a biomarker of DNA oxidative damage. PM can promote DNA damage *in vitro* via 8-OHdG formation and strand breaks 119, 120. Interestingly, genotoxicity is also an important mechanism by which the lung microbiome affects lung cancer (as mentioned before), which may be the key to linking the three. Otherwise, DNA oxidative damage can also be caused by ROS and reactive nitrogen species (RNS) that are excessively formed by inflammatory cells during the particle-elicited inflammation process 121. Microorganisms are associated with cancer-inducing inflammation 51. However, the exact inflammatory cytokines and cells need to be identified to confirm this possible relationship.

Abnormal promoter methylation of tumor suppressor genes is another possible link between air pollution-related microbiome changes and lung cancer. Continuous PM2.5 exposure induced methylation of the tumor suppressor gene p53 promoter by promoting methylation of DNA (cytosine-5-)-methyltransferase 3β, thereby leading to inactivation of p53 in human alveolar epithelial cells 122. Particulate carcinogens cause lung cancer in rats, half of which show p16 methylation 123. Microorganisms are also related to aberrant DNA methylation 124, but the role of the microbiome in DNA methylation changes associated with air pollution needs to be verified by further research.

The specific status of respiratory microorganisms in the effect of air pollution on lung cancer needs to be additionally explored. Increasing research in this field has found novel directions for tumor prevention and treatment. For example, vitamins B6 and C can partially attenuate the gene-specific hypermethylation and hypomethylation caused by B[a]P 125, which may have preventive effects on air pollution-related lung cancer.

## Prospective

It is expected that related research will further clarify the relationship between the respiratory microbiome and respiratory tumors, provide clues for exploring new tumor therapies and developing antitumor drugs targeting microbes, and promote the rational use of angiogenesis inhibitors in antitumor therapy from the perspective of the human microbiota. At the same time, the abnormal structure of the tumor blood vessels mentioned above will reduce the various treatment methods to reach the tumor tissue, thereby reducing the treatment efficiency of the tumor. Therefore, perhaps we can propose a different idea and perspective for tumor treatment: improving abnormal angiogenesis of the tumor will limit the hypoxia of the tumor and prevent the selective effect of hypoxia on the highly invasive tumor.

## Figures and Tables

**Figure 1:**
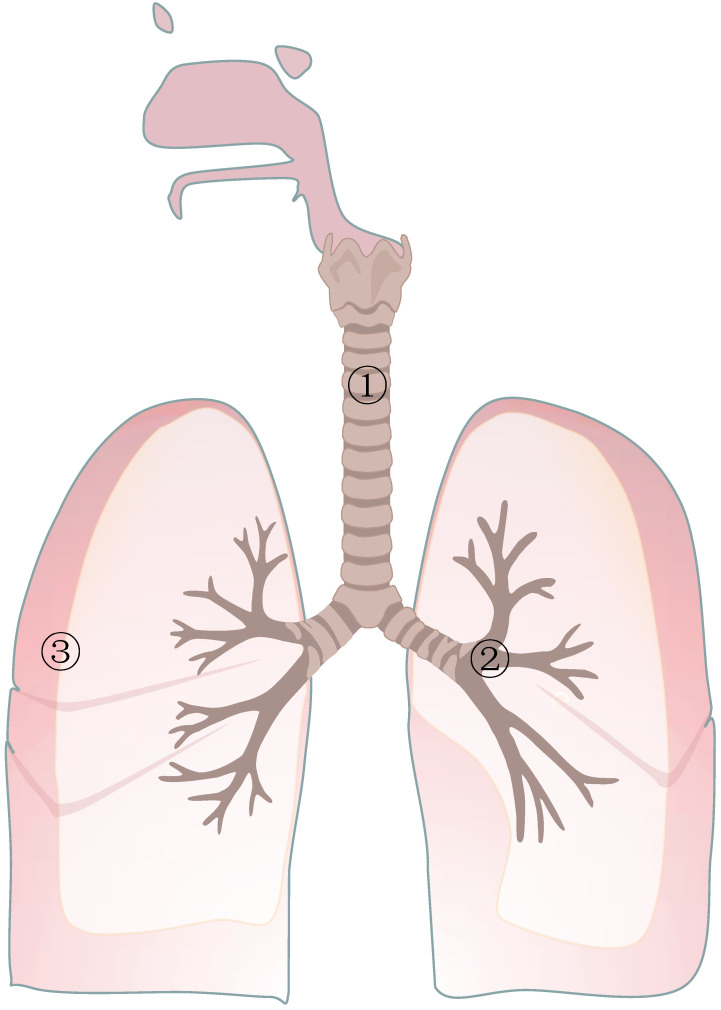
Representative bacteria in different circumstances. In the normal respiratory tract, the representative phyla and classes are *Proteobacteria* and* Firmicutes*, and the representative genera are *Pseudomonas* and *Streptoccus*, respectively. In the respiratory tract with lung cancer, some bacteria tend to be less abundant, such as *Propionibacterium* and *Enterobacteriaceae*, and some tend to be more abundant, such as *M.tuberculosis* and *Helicobacter*. In the lungs of healthy people, the most common bacterial microorganisms are* Firmicutes*, *Bacteroidetes*, *Proteobacteria*, *Actinobacteria*, and *Fusobacterium*. In the lungs of patients with lung cancer, *Atopobium* and *Selenomonas* were obviously decreased. In contrast, additional microorganisms were* Firmicutes*, *TM7*, *Veillonella*, *Megasphaera*, *Filifactor* and *Selenomonas*.

**Table 1:** Representative bacteria in different circumstances

		Respiratory Tract ①,②	Lung③
Normal		Phylum and class: *Proteobacteria, Firmicutes, Fusobacteria, Bacteroidetes.* Genus: * Pseudomonas, Streptococcus, Prevotella, Veillonella, Haemophilus, Neisseria.*	*Firmicutes, Bacteroidetes, Proteobacteria, Actinobacteria, Fusobacterium.*
Cancer	Less	*Propionibacterium, Enterobacteriaceae, Prevotella, Pseudobutyrivibrio, ruminis.*	*Atopobium*
			*Selenomonas*
	More	*M.tuberculosis, Helicobacter, Pylori, Actinomyces, Peptostreptococcus, Streptococcus, Wechsler, Modestobacter, Eubacterium xylanophilum, Eubacterium Eligens, Clostridium.*	Phylum:
			*Firmicutes*
			*TM7*
			Genus:
			*Veillonella, Megasphaera Filifactor Selenomonas*

## Abbreviations

IL-17: interleukin-17
H. Pylori: Helicobacter pylori
F. nucleatum: Fusobacterium nucleatum
HPV: human papilloma virus
AMs: alveolar macrophages
TAMs: tumor-associated macrophages
M. tuberculosis: Mycobacterium tuberculosis
SCLC: small cell lung cancer
BALF: bronchoalveolar lavage fluid
OTUs: operational taxonomic units
EBV: Epstein-Barr virus
EBER: EBV-encoded small nonpolyadenylated RNA
LELC: lymphoepithelioma-like carcinoma
LMP1: latent membrane protein 1
VCA: viral capsid antigen
LSH: lymphoid-specific helicase
NSCLC: non-small cell lung cancer
GINS4: Go, Ichi, Nii, and San complex subunit 4
HIV: human immunodeficiency virus
JC virus: John Cunningham virus
HMP: the Human Microbiome Project
PRR: pattern-recognition receptor
CD: cluster of designation
NTHi: nontypeable Haemophilus influenza
IL-6: interleukin-6
NK cell: natural killer cell
PARP1: poly (ADP-ribose) polymerase 1
ROS: reactive oxygen species
PTPA: protein tyrosine phosphatase A
VEGF: vascular endothelial growth factor
Th17: T helper type 17
ECs: endothelial cells
TLR: toll-like receptor
DLL4: delta-like 4
IL-8: interleukin-8
GPCRs: G protein-coupled receptors
MetAP2: methionine aminopeptidase 2
BM: basement membrane
ECM: extracellular matrix
MMP: matrix metalloprotease
EPC: endothelial progenitor cell
PDGF: platelet derived growth factor
HGF: hepatocyte growth factor
FGF: fibroblast growth factor
VEGFR: vascular endothelial growth factor receptor
CAF: cancer-associated fibroblasts
NO: nitric oxide
TGF-β: transforming growth factor-β
COPD: chronic obstructive pulmonary disease
HIF-1α: hypoxia-inducible factor-1α
ICBs: immune checkpoint blockers
CTLA-4: cytotoxic lymphocyte antigen-4
PD-L1: programmed cell death protein 1 ligand 1
IL-12: interleukin-12
PM: particulate matter
HAP: household air pollution
B[a]P: benzo[a]pyrene
8-OHdG: 8-hydroxy-2'-deoxyguanosine
RNS: reactive nitrogen species
